# Integration analysis of microRNA and mRNA paired expression profiling identifies deregulated microRNA-transcription factor-gene regulatory networks in ovarian endometriosis

**DOI:** 10.1186/s12958-017-0319-5

**Published:** 2018-01-22

**Authors:** Luyang Zhao, Chenglei Gu, Mingxia Ye, Zhe Zhang, Li’an Li, Wensheng Fan, Yuanguang Meng

**Affiliations:** 1 Department of Gynecology and Obstetrics, People’s Liberation Army (PLA) Medical School, Chinese PLA General Hospital, Beijing, 100853 China; 2 Department of Gynecology and Obstetrics, Peking University People’s Hospital, Beijing, China; 3 Department of Gynecology and Obstetrics, the 309th Hospital of Chinese PLA, Beijing, China

**Keywords:** Endometriosis, Endometrium, MicroRNAs, Transcription factors, Integrative analysis

## Abstract

**Background:**

The etiology and pathophysiology of endometriosis remain unclear. Accumulating evidence suggests that aberrant microRNA (miRNA) and transcription factor (TF) expression may be involved in the pathogenesis and development of endometriosis. This study therefore aims to survey the key miRNAs, TFs and genes and further understand the mechanism of endometriosis.

**Methods:**

Paired expression profiling of miRNA and mRNA in ectopic endometria compared with eutopic endometria were determined by high-throughput sequencing techniques in eight patients with ovarian endometriosis. Binary interactions and circuits among the miRNAs, TFs, and corresponding genes were identified by the Pearson correlation coefficients. miRNA-TF-gene regulatory networks were constructed using bioinformatic methods. Eleven selected miRNAs and TFs were validated by quantitative reverse transcription-polymerase chain reaction in 22 patients.

**Results:**

Overall, 107 differentially expressed miRNAs and 6112 differentially expressed mRNAs were identified by comparing the sequencing of the ectopic endometrium group and the eutopic endometrium group. The miRNA-TF-gene regulatory network consists of 22 miRNAs, 12 TFs and 430 corresponding genes. Specifically, some key regulators from the miR-449 and miR-34b/c cluster, miR-200 family, miR-106a-363 cluster, miR-182/183, FOX family, GATA family, and E2F family as well as CEBPA, SOX9 and HNF4A were suggested to play vital regulatory roles in the pathogenesis of endometriosis.

**Conclusion:**

Integration analysis of the miRNA and mRNA expression profiles presents a unique insight into the regulatory network of this enigmatic disorder and possibly provides clues regarding replacement therapy for endometriosis.

**Electronic supplementary material:**

The online version of this article (10.1186/s12958-017-0319-5) contains supplementary material, which is available to authorized users.

## Background

Endometriosis is characterized by the presence of endometrial tissue (endometrial glandular and stromal) abnormally outside the uterine cavity [1]. In women of reproductive age, there is a 6–10% incidence rate, which may even reach 35–50% in women who are enduring pelvic pain and/or infertility [2, 3]. Despite its high prevalence, the etiology and pathophysiology of endometriosis are still not fully understood. It is widely accepted that endometriosis is a multifactorial, polygenic disorder [4]. A large amount of molecular aberrations exist between the ectopic endometrium (EC) (endometriotic lesions) and the eutopic endometrium (EU), which could potentially explain the mechanism of the abnormal growth of EU outside the uterus.

microRNAs (miRNAs) are endogenous, single-stranded, 18–22 nucleotide RNAs that mainly inhibit gene expression at the post-transcriptional level [5]. They negatively regulate the translation of target messenger RNAs (mRNAs) via the sequence specific recognition of the “seed sequence” and repress translation and/or degrade the target mRNAs according to the degree of complementary nucleotides [6]. Transcription factors (TFs) are a group of proteins that regulate gene expression by binding to specific DNA sequences at the transcriptional level [7]. Depending on its function, the transcription of an adjacent gene is either activated or repressed [8]. As two vital gene regulatory molecules, miRNAs and TFs can share common target genes and exert effects on each other [9]. As such, the miRNA expression levels can be regulated by TFs, and the mRNAs encoding TFs can be inhibited by miRNAs [10]. In addition, miRNAs and TFs can form a feed-back loop (FBL) or feed-forward loop (FFL) in cooperation to fine tune gene expression [11]. The combinatorial regulation of TFs and miRNAs is suggested to play vital roles in various biological processes and disease pathogenesis [12–15].

Accumulating evidence suggests that aberrant miRNA and TF expression may be involved in the pathogenesis and development of endometriosis [16–25]. Although differences in miRNA expression profiling between EC and EU from women with endometriosis have previously been reported, almost no previous studies have focused on the integrative analysis of miRNA-mRNA interactions and solved the complex problem of the identification of miRNA-TF-gene regulatory networks. With the aim of further understanding the abnormal molecule changes in the occurrence and development of endometriosis, we used high-throughput sequencing techniques to detect miRNA and mRNA expression profiling in paired EC and EU. Joint analysis of the miRNA-TF-gene regulatory network can help us survey the key genomic factors and understand the mechanism of endometriosis at the molecular level.

## Methods

### Patient samples

The study protocol was approved by the Local Ethical Committee of the Chinese PLA General Hospital, and each patient was required to provide written consent.

Sixty tissue samples (30 paired EC and EU) from 30 patients with ovarian endometriosis were obtained from the Chinese PLA general hospital. Among them, eight paired EC and EU samples were selected and prepared for small RNA sequencing and mRNA sequencing. The remaining samples were used for validation. All of the patients were confirmed to have endometriosis by histological examination and diagnosed as being at a moderate to severe (III-IV) stage by the revised American Fertility Society (r-AFS) classification during laparoscopic surgery [26]. EC samples were obtained during laparoscopy, and EU samples were obtained through curettage before the laparoscopic procedure. Only patients in the secretory phase of the menstrual cycle, which was confirmed by the method of Noyes et al. [27], and without any hormonal treatment history, were included in the study. Clinical characteristics of the patients are listed in Additional file 1.

### Tissue processing, RNA extraction and quality control

All tissue samples were divided into two parts: one half was fixed and prepared for pathological examination and the other half was placed in RNA-later solution (Sigma Aldrich, Poland) at 4 °C for 24 h and subsequently transferred to −80 °C until further use. Total RNA was extracted using a single-step acid guanidinium thiocyanate-phenol-chloroform method [28]. The quality and purity of RNA were examined by a Nanodrop 8000 spectrophotometer (Thermo Scientific, Waltham, MA, USA). RNA integrity was analyzed using the RNA 6000 Nano Kit and Small RNA Kit with the Bioanalyzer 2100 (Agilent, Santa Clara, CA). Samples with an absorbance wavelength ratio (A260/A280) ≥ 1.9 and an RNA integrity number ≥ 8 were included.

### Small RNA sequencing (small RNA-seq) and data analysis

For small RNA sequencing, 3 μg of total RNA was used for library preparation as previously reported with little modification [29]. The purified libraries were sequenced on an Illumina HiSeq 2000 platform by Annoroad Genome (Beijing, China). The detailed protocol is described in Additional file 2. In brief, the raw data were processed with Python scripts to ensure its quality and were then filtered by Q30 statistics. The clean data were mapped to the Ensemble database (GRCh37) (http://grch37.ensembl.org/index.html) [30] using Bowtie (v1.01) [31] and compared with miRBase (Release 21) [32] to identify mature miRNAs. For each sample, the count and RPM (reads per million total reads) value of the miRNAs were collected. DEGseq (v1.18.0) [33] was used for differential expression analysis with the parameters of differentially expressed miRNAs (DEM) set with a false discovery rate (FDR) < 0.05 and |log2 fold change (FC)| ≥ 1.

### mRNA sequencing (mRNA-seq) and data analysis

For mRNA sequencing, 3 μg of RNA per sample was prepared for library preparation. The Ribo-Zero Gold Kit (Epicentre, USA) and NEB Next Ultra RNA Library Prep Kit (NEB, Ipswich, USA) were used for rRNA removal and library construction following the manufacturer’s protocols. For high-throughput sequencing, paired-end 150-bp sequencing of the cDNAs was performed on an Illumina HiSeq4000 system (Illumina, USA) conducted by Annoroad Genome (Beijing, China). The raw data were processed with Perl scripts to ensure the quality of the data used in subsequent analyses (details in Additional file 2). Bowtie2 (v2.2.3) [34] was used to build the genome index, and clean data were mapped to the human genome (GRCh37) (http://grch37.ensembl.org/index.html) using TopHat (v2.0.12) [35]. The read counts of each gene were counted by HTSeq (v0.6.0) [36], and the reads per kilobase of one gene per million reads (RPKM) were calculated to estimate the expression level of genes in each sample. DEGseq (v1.18.0) [33] was used to analyze differentially expressed genes (DEGs) with parameters of FDR < 0.05 and |log2FC| ≥ 1.

### Identification of miRNA/TF/gene interactions and a regulatory network

The miRNA and mRNA expression profiles were analyzed by MAGIA2 (http://gencomp.bio.unipd.it/magia2/) to identify miRNA-TF-gene binary interactions and circuits [37]. The miRNA-target interactions were predicted by the TargetScan database [38] with a z-score ≥ 0.7. TF-miRNA interactions were identified from the mirGen2.0 [39] and TransmiR [40] databases, and TF-gene interactions were obtained from the ‘TFBS conserved’ track of the UCSC genome annotation for human (version hg19) (http://genome.ucsc.edu/) database, restricting the z-score to ≥ 3. Two types of mixed regulatory circuits were also identified: (i) FFL, which is a TF that regulates both a given miRNA and their common target gene and (ii) FBL, which is a miRNA that regulates both a given TF and their common regulated gene. The Pearson correlation coefficient (PCC) was used to measure the relationships with a PCC > 0.6 and a *P*-value < 0.05. The integrative miRNA-TF-gene regulatory network was visualized by Cytoscape (v3.2) [41].

### Functional analysis

To explore the functional roles of DEGs in the miRNA-TF-gene networks, we used DAVID, which integrates the Gene Ontology (GO) and Kyoto Encyclopedia of Genes and Genomes (KEGG) databases to analyze biological functions [42]. The enrichment values of GO terms and KEGG pathways were implemented by the hypergeometric test, and q (adjusted as *p*-value) < 0.05 was considered to be significantly enriched.

### Quantitative reverse transcription-polymerase chain reaction (qRT-PCR) validation

Six miRNAs (miR-34c-5p, miR-106a-5p, miR-182-5p, miR-200a-3p, miR-449b-5p, and miR-615-3p) and five TFs (CEBPA, FOXC1, E2F1, GATA1, and HNF4A) were selected for validation analysis. U6 was selected as the miRNA endogenous control, and GAPDH was used as the mRNA endogenous control. Primer sequences are listed in Additional file 3. miRNA cDNA synthesis was conducted with the miRNA cDNA Kit (ComWin Biotech, Beijing), and total cDNA synthesis was conducted by a RevertAid™ First Strand cDNA Synthesis Kit (Thermo, USA). Relative miRNA expression was determined according to the miRNA Real-Time PCR Assay Kit (ComWin Biotech, Beijing), and relative mRNA expression was determined according to the THUNDERBIRD™ SYBR qPCR Mix (TOYOBO, Japan). qRT-PCR was performed on an ABI PRISM 7500 (Applied Biosystems, CA, USA). Relative expression was calculated using the ABI PRISM 7500 version 2.0.6 software (Applied Biosystems, CA, USA) with the 2^−ΔΔCt^ method [43].

## Results

### Small RNA-seq analysis and DEM profiling

**Fig. 1:**
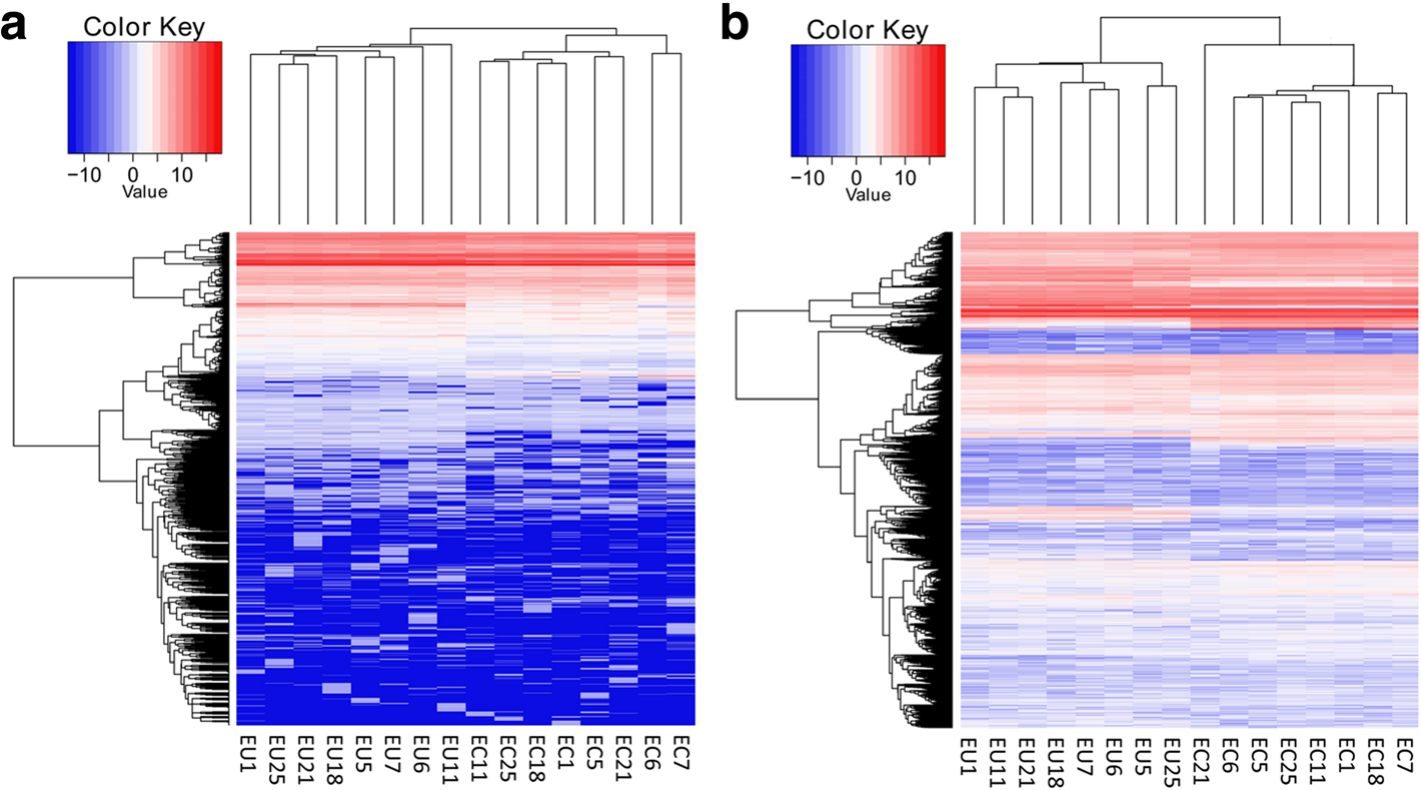
Heat map showing differentially expressed miRNAs and mRNAs from ectopic endometria compared with paired eutopic endometria. Rows represent miRNAs (**a**) or mRNAs (**b**); columns represent profiled samples. The relative expression is depicted according to the color scale. Red indicates up-regulation; blue indicates down-regulation. EU means eutopic endometrium and EC means ectopic endometrium

The small RNA-seq generated 216,333,583 reads with an average of 13,520,849 reads per sample. The FastQC quality test showed that 182,420,380 of the reads had a Q-score ≥ 30; these reads were considered in further analyses. A proportion of clean reads was mapped to the miRBase database (v21.0) for each sample and is listed in Additional file 4. Of the 1574 miRNAs analyzed by small RNA sequencing, 1163 with a normalized count value greater than 1 in at least one sample in both the EC and EU groups were further analyzed. Unsupervised hierarchical clustering showed that the overall miRNA profile of EC was wholly different from that of EU (Fig.1a). Using the selection criteria mentioned above, 107 DEMs were found, including 41 up-regulated miRNAs and 66 down-regulated miRNAs, between EC and paired EU. Among them, miR-514b-3p was the most up-regulated, with an FC of 330.31, whereas miR-375 was the most down-regulated, with an FC of 154.52 (Additional file 5).

### mRNA-seq analysis and DEG profiling

mRNA-seq generated 1,748,534,484 reads with an average of 109,283,405 reads per sample. The FastQC quality test showed that 1,721,709,042 (98.46%) reads reached Q30; these reads were considered for further analyses. Of these reads, 93.17% were mapped to hg19, and 98.56% were uniquely aligned. The detailed filtering and mapping data are listed in Additional file 6. Unsupervised hierarchical clustering analysis showed that the transcriptome profiling of the EC group was significantly different from that of the EU group (Fig. 1b). Using the same criteria that were used for the miRNAs, a total of 6112 DEGs with 3228 up-regulated mRNA transcripts and 2884 down-regulated mRNA transcripts were identified. The most up- and down-regulated mRNAs were IGHV3–49 (ENSG00000211965) and FXYD4 (ENSG00000150201), with FCs of 3253.41 and 1024.16, respectively. Additional file 7 lists the top 25 up- and down-regulated mRNAs identified by our sequencing.

### Identification of miRNA/TF/gene interactions and regulatory networks

One hundred seven DEMs and 6112 DEGs identified in the previous steps were considered for the miRNA/TF/gene binary interaction and regulatory network analysis.

According to the standard of absolute PCC > 0.6, correlation analysis revealed 379 paired miRNA-target regulatory interactions between 15 miRNAs (miR-20b-5p, miR-30d-5p, miR-34c-5p, miR-106a-5p, miR-133b, miR-141-3p, miR-182-5p, miR-183-5p, miR-196b-5p, miR-200a-3p, miR-200b-3p, miR-200c-3p, miR-363-3p, miR-449a, and miR-449b-5p), five TFs (GATA6, GATA2, FOXO1, FOXC1, and CEBPA) and 223 genes. Although miRNAs usually suppress target mRNAs, positively paired miRNA-mRNA correlations are occasionally observed under certain conditions [44]. For this reason, we identified all of the differentially expressed miRNA-gene pairs, including 174 negative correlating pairs and 205 positive correlating pairs. In the case of TF-gene interactions, 310 regulatory pairs involving seven TFs (E2F1, CEBPA, FOXC1, FOXL1, FOXO1, GATA1, and SOX9) and 261 corresponding genes were identified. For TF-miRNA interactions, a total of 23 TF-miRNA pairs between seven TFs (E2F1, E2F2, FOXJ1, FOXL1, GATA1, HNF4A, and SOX9) and 19 miRNAs (miR-20b-5p, miR-34b-3p, miR-34b-5p, miR-34c-5p, miR-106a-5p, miR-133b, miR-182-5p, miR-183-5p, miR-196b-5p, miR-200a-3p, miR-200b-3p, miR-202-3p, miR-216a-5p, miR-224-5p, miR-363-3p, miR-449a, miR-449b-5p, miR-449c-5p, and miR-708-5p) were involved in the comparison. Moreover, we revealed five mixed circuits, including one FFL (FOXL1/miR-133b/PRDM1) and four FBLs (miR-182-5p/FOXO1/NUAK1, miR-182-5p/FOXO1/SCN9A, miR-133b/FOXC1/EYA4, and miR-133b/FOXC1/LHFP).

**Fig. 2:**
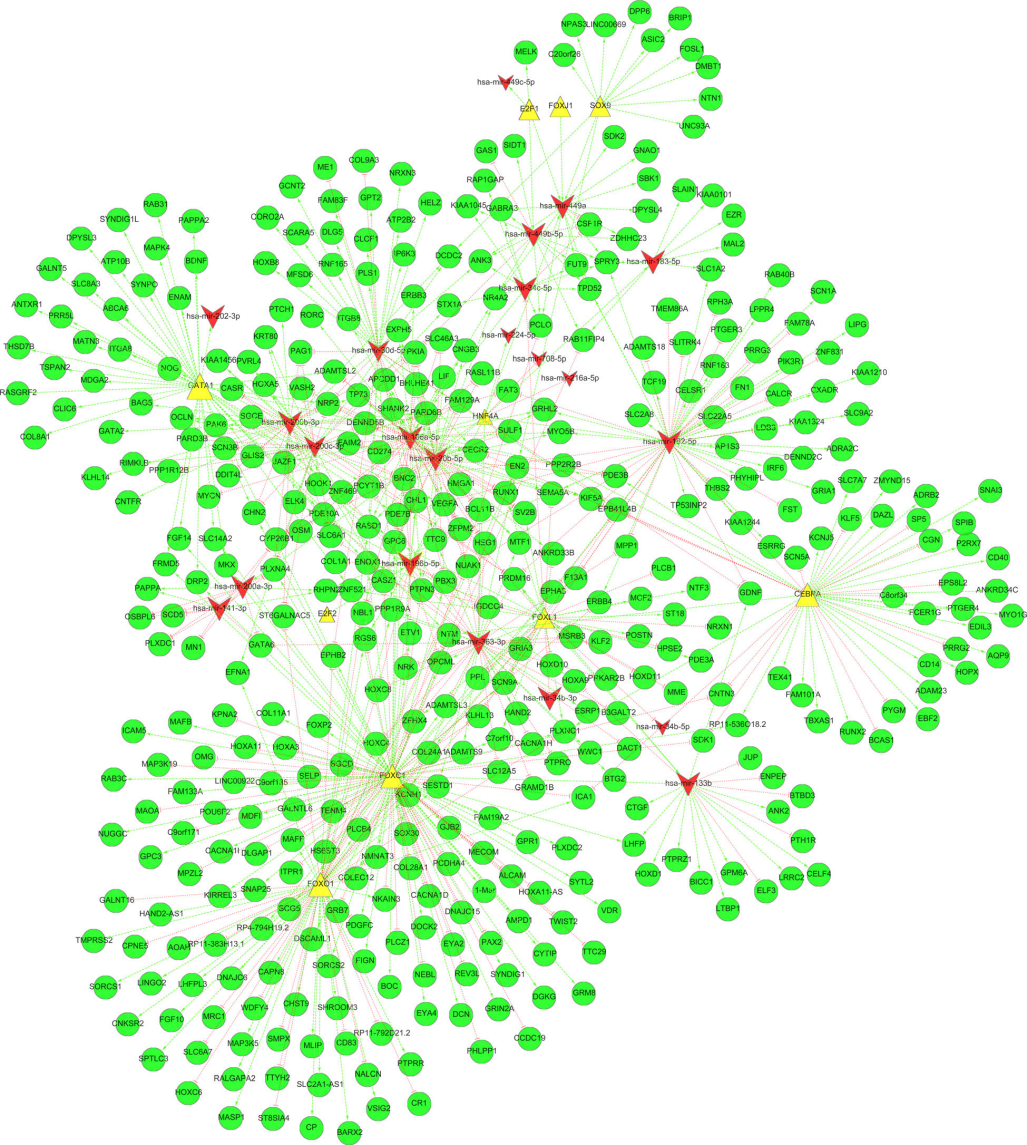
The integrated endometriosis-related miRNA-transcription factor-gene regulation network. Red represents miRNA; yellow represents transcription factor; green represents target gene

Based on the relationships of binary interactions and circuits, we constructed the miRNA-TF-gene regulatory network. The final integrated network contained 22 miRNAs, 12 TFs and 430 genes (Table 1, Fig. 2). To identify key regulators in the integrated networks, we also calculated the node degree of the involved miRNAs and TFs. The node degree is a centrality measure method that directly counts the links of one node. The few nodes that have a high degree in the network are called hubs, which reflect their importance in a large number of interactions [45]. As a result, the node degrees of the selected miRNAs and TFs ranged from 1 to 117 in our data. The top five key regulators in the miRNA-TF-gene network of endometriosis were FOXC1, FOXO1, miR-182-5p, miR-106a-5p, and CEBPA, which were directly connected to 117, 64, 57, 49, and 49 corresponding targets, respectively (Table 1).

**Table 1:** The key regulators and their targets identified in the integrated network in endometriosis

ID	Node degree	Fold change	Target
FOXC1	117	7.26	ZNF521, MKX, ENOX1, TENM4, CHN2, PLXDC2, ZFHX4, COLEC12, FAM19A2, ICAM5, DCN, DGKG, PTPN3, PDGFC, PBX3, EPHA3, C7orf10, NMNAT3, MDFI, SCG5, GPC6, FGF14, ADAMTSL3, F13A1, FIGN, SNAP25, BNC2, GPR1, ICA1, BOC, PLXNC1, NRK, VDR, OMG, CHL1, SYNDIG1, DRP2, EPHB2, SORCS2, RP4-794H19.2, SDK1, PLCB4, HS6ST3, NTM, FAM133A, HOXA11, NUGGC, SELP, TTC29, HOXA3, NKAIN3, SYTL2, HOXA11-AS, EN2, CNTN3, DNAJC15, GPC3, GALNTL6, LINC00922, CASZ1, COL11A1, RAB11FIP4, RAB3C, AMPD1, C9orf135, TWIST2, MAP3K19, C9orf171, FRMD5, GRM8, GJB2, MECOM, ETV1, ALCAM, DOCK2, REV3L, EYA4, CACNA1D, PLXNA4, GRB7, SCN9A, PDE7B, PAX2, DLGAP1, PCDHA4, KPNA2, KIRREL3, CCDC19, SLC14A2, KLHL13, COL28A1, MAOA, MARCH1, CACNA1I, NEBL, SGCD, MAFB, LHFP, DSCAML1, ADAMTS9, POU6F2, PHLPP1, EYA2, ERBB4PCLO, OPCML, SESTD1, MAFF, MPZL2, SOX30, PLCZ1, RP11-536O18.2, RGS6, GRIN2A, CYTIP, ITPR1
FOXO1	64	−3.56	HAND2-AS1, EFNA1, GRB7, RASD1, EN2, ICA1, SESTD1, CNKSR2, RP11-383H13.1, VSIG2, CP, RP11-792D21.2, MRC1, PDGFC, BARX2, KCNH1, NKAIN3, HS6ST3, CHST9, WDFY4, HOXC4, PPP1R9A, NUAK1, PTPRR, LHFPL3, CR1, SHROOM3, PLCB4, RGS6, HOXC8, TMPRSS2, LINGO2, AOAH, COLEC12, MASP1, MAP3K5, MLIP, SPTLC3, TENM4, SLC2A1-AS1, PLXNC1, ZFHX4, SLC6A7, RALGAPA2, SORCS1, EPHB2, DNAJC6, HOXC6, MAFF, NMNAT3, NALCN, SMPX, SCN9A, ST8SIA4, CAPN8, TTYH2, NBL1, CD83, GALNT16, NTM, COL24A1, ST6GALNAC5, CPNE5, FGF10
miR-182-5p	57	−5.34	RAB40B, SLC1A2, SCN1, APHYHIPL, FAT3, SLC2A8, **FOXO1**, **CEBPA**, PDE7B, RPH3A, ESRRG, CALCR, RHPN2, TP53INP2, SLITRK4, PTGER3, FN1, DENND2C, EPHA3, ZNF831, PIK3R1, SLC9A2, TCF19, SCN9A, GRIA1, FUT9, LDB3, PRRG3, PRDM16, SPRY3, ADRA2C, THBS2, SLC22A5, LIPG, FST, BNC2, ADAMTS18, GDNF, MPP1, FAM78A, KIAA1210, TMEM86A, RNF183, F13A1, ERBB4, NUAK1, IRF6, SEMA5A, LPPR4, AP1S3, MYO5B, KIAA1244, TPD52, GRHL2, CXADR, CELSR1, KIAA1324
miR-106a-5p	49	−3.72	VASH2, OSM, MYCN, JAZF1, ETV1, BCL11B, TP73, ITGB8, SV2B, CD274, ANKRD33B, GRHL2, DENND5B, CYP26B1, LIF, PKIA, ERBB3, RASL11B, RORC, VEGFA, NR4A2, SHANK2, PARD6B, BHLHE41, GPC6, RASD1, PDE3B, HEG1, RUNX1, SULF1, MYO5B, TTC9, CNGB3, ZFPM2, BNC2, SLC46A3, PBX3, EXPH5, ADAMTSL2, PCYT1B, APCDD1, FAM129A, SEMA5A, FAIM2, MTF1, KIF5A, NBL1, PTPN3, NRP2
CEBPA	49	5.73	VASH2, OSM, MYCN, JAZF1, ETV1, BCL11B, TP73, ITGB8, SV2B, CD274, ANKRD33B, GRHL2, DENND5B, CYP26B1, LIF, PKIA, ERBB3, RASL11B, RORC, VEGFA, NR4A2, SHANK2, PARD6B, BHLHE41, GPC6, RASD1, PDE3B, HEG1, RUNX1, SULF1, MYO5B, TTC9, CNGB3, ZFPM2, BNC2, SLC46A3, PBX3, EXPH5, ADAMTSL2, PCYT1B, APCDD1, FAM129A, SEMA5A, FAIM2, MTF1, KIF5A, NBL1, PTPN3, NRP2
GATA1	41	7.62	**miR-202-3p**, ATP10B, MATN3, PAPPA2, RAB31, PDE10A, FRMD5, ABCA6, PRR5L, TSPAN2, SLC8A3, SYNDIG1L, MDGA2, ENOX1, FOXC1, FGF14, CLIC6, RASGRF2, ANTXR1, ZNF469, PCYT1B, MAPK4, ENAM, NRP2, SYNPO, DRP2, CHN2, PDE7B, ZNF521, PPP2R2B, ITGA8, NTM, MKX, SLC14A2, GALNT5, COL8A1, BDNF, ADAMTSL2, DPYSL3, THSD7B, GPC6
miR-20b-5p	40	−4.16	TP73, LIF, CYP26B1, RASL11B, GRHL2, SV2B, RASD1, CD274, DENND5B, VEGFA, HEG1, BCL11B, PARD6B, PKIA, MYO5B, RUNX1, GPC6, BHLHE41, PDE3B, SULF1, SHANK2, TTC9, EXPH5, NR4A2, SLC46A3, ZFPM2, BNC2, PCYT1B, ADAMTSL2, NBL1, PBX3, APCDD1, FAM129A, FAIM2, KIF5A, MTF1, PTPN3, NRP2, SEMA5A, CNGB3
miR-200b-3p	37	−16.72	PLXNA4, CASR, ELK4, PARD3B, DCDC2, DENND5B, PARD6B, PKIA, KRT80, HOXA5, KLHL14, SGCE, NOG, PTCH1, **GATA2**, ST6GALNAC5, PRDM16, BNC2, SLC6A1, TP73, SCN3B, ANK3, HOOK1, PAG1, PVRL4, BAG5, GLIS2, JAZF1, DDIT4L, CNTFR, CHN2, CASZ1, NRP2, KIAA1456, OCLN, CECR2, PAK6
FOXL1	37	20.45	**miR-34c-5p, miR-34b-3p, miR-34b-5p, miR-133b**, PDE3A, SV2B, PRDM16, HOXD10, ZFHX4, PLXNC1, MSRB3, ZNF521, SEMA5A, HOXD11, ST18, SCN9A, MCF2, ENOX1, NTF3, FOXO1, OPCML, EPB41L4B, C7orf10, PPL, KLHL13, CASZ1, MME, POSTN, RUNX1, COL24A1, VEGFA, GPC6, ANKRD33B, HPSE2, GRIA3, NRXN1, PLCB1
miR-363-3p	36	−2.92	EN2, SLC12A5, NRK, BCL11B, SESTD1, PRKAR2B, DACT1, PTPRO, RHPN2, HEG1, BTG2, CACNA1H, B3GALT2, PPP1R9A, ZNF469, PRDM16, KLF2, HAND2, ZFHX4, ESRP1, WWC1, GRAMD1B, SLC6A1, MPP1, ZFPM2, **GATA6**, ADAMTSL3, TTC9, ADAMTS9, PDE10A, PCYT1B, MSRB3, HOXC8, HOXC4, MTF1, KCNH1
miR-200c-3p	36	−27.15	PPP1R12B, CASR, FAT3, STX1A, DCDC2, VEGFA, PLXNA4, VASH2, PARD3B, JAZF1, PKIA, KRT80, DENND5B, RIMKLB, HOXA5, KIAA1456, SGCE, SLC6A1, PAK6, PRDM16, SCN3B, ST6GALNAC5, BNC2, HOOK1, ELK4, EFNA1, CASZ1, GLIS2, DDIT4L, PVRL4, PAG1, NRP2, CHN2, CECR2, PARD6B, OCLN
miR-30d-5p	29	−3.93	CHL1, MFSD6, CLCF1, PLS1, COL9A3, BNC2, NRXN3, ME1, EPHB2, NUAK1, PAG1, NRP2, RGS6, HOXB8, GDNF, CORO2A, EPB41L4B, RASD1, PPP1R9A, GCNT2, HELZ, ATP2B2, CECR2, GPT2, IP6K3, SCARA5, DLG5, RNF165, FAM83F
miR-133b	19	9.16	BTBD3, FOXC1, ENPEP, JUP, ELF3, PTH1R, PRDM16, GPM6A, CTGF, BICC1, HOXD1, HOXA9, LHFP, CELF4, LTBP1, GDNF, PTPRZ1, LRRC2, ANK2
miR-449b-5p	14	−114.1	PKIA, FUT9, GAS1, NR4A2, RAB11FIP4, CSF1R, KIAA1045, ZDHHC23, GABRA3, SIDT1, ANK3, SPRY3, TPD52, PCLO
miR-449a	13	−70.83	CSF1R, ANK3, ZDHHC23, FUT9, SDK2, GNAO1, GABRA3, TPD52, SBK1, NR4A2, SPRY3, PCLO, DPYSL4
miR-196b-5p	13	−13.72	HMGA1, OPCML, COL1A1, NRK, IGDCC4, RGS6, ELK4, PARD6B, HOOK1, BNC2, PBX3, HOXA9, HOXC8
SOX9	13	−12.89	MELK, DCDC2, NTN1, DPP6, UNC93A, FOSL1, ASIC2, LINC00669, NPAS3, BRIP1, DMBT1, EN2, C20orf26
HNF4A	12	−9.63	**miR-20b-5p, miR-363-3p, miR-106a-5p, miR-196b-5p, miR-182-5p, miR-224-5p, miR-183-5p, miR-200a-3p, miR-708-5p, miR-200b-3p, miR-216a-5p, miR-449a**
miR-34c-5p	11	−15.91	SPRY3, KIAA1045, GABRA3, ANK3, STX1A, CSF1R, PARD6B, PCLO, RAP1GAP, TPD52
miR-200a-3p	9	−35.83	SCD5, PLXNA4, CYP26B1, PLXDC1, PAPPA, OSBPL6, MN1, RHPN2, GATA6
miR-141-3p	9	−56.69	PLXNA4, PLXDC1, SCD5, PAPPA, MN1, CYP26B1, GATA6, OSBPL6, RHPN2
miR-183-5p	7	−5.72	EZR, RAB11FIP4, KIAA0101, SLC1A2, MAL2, SLAIN1, SPRY3
E2F1	4	−3.74	**miR-449a, miR-449c-5p, miR-449b-5p**, MELK
FOXJ1	1	−98.82	**miR-449a**
E2F2	1	−3.56	**miR-196b-5p**

The identified miRNAs and TFs in “Target” were highlighted in bold type

### Functional analysis

**Fig.3:**
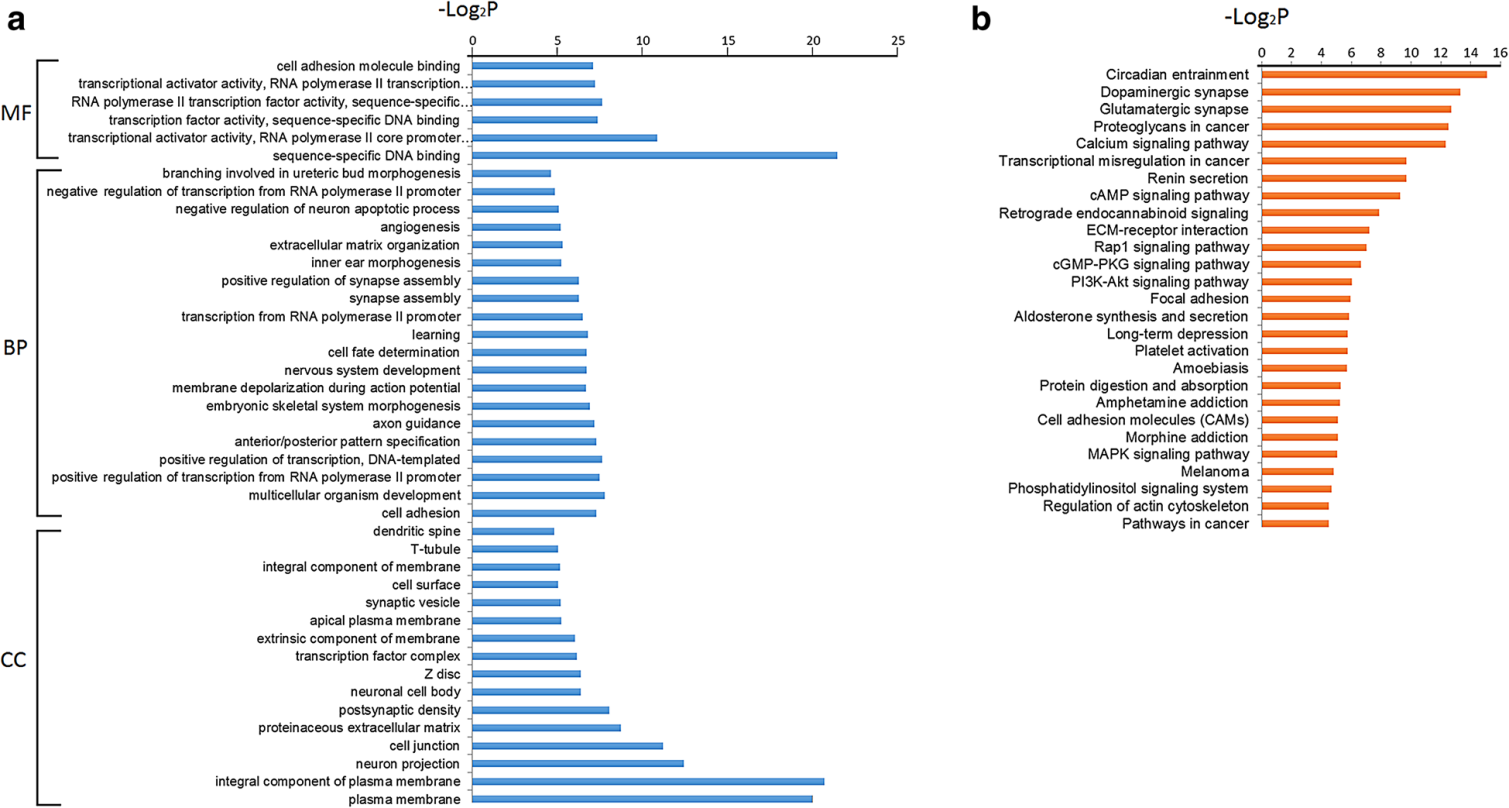
a GO terms enriched by differentially expressed genes that involved in regulatory network. BP represents biological processes; CC represents cellular components; MF represents molecular functions. b KEGG pathways enriched by differentially expressed genes that involved in regulatory network

Functional enrichment analysis of all of the target genes involved in the networks provided us with an overall clue regarding their functional roles in endometriosis. GO analysis revealed that the target genes in the GO cellular component (GO-CC) category were enriched in 16 terms, such as the plasma membrane, proteinaceous extracellular matrix and Z disc. The GO molecular function (GO-MF) category was enriched in six terms, including sequence-specific DNA binding, transcriptional activator activity and cell adhesion molecule binding. In the biological process category of GO terms (GO-BP), 20 terms included cell adhesion, whereas embryonic skeletal system morphogenesis, extracellular matrix organization and angiogenesis were significantly enriched (Fig. 3a). KEGG pathway analysis demonstrated that the DEG targets were associated with 27 pathways, such as the calcium-signaling pathway, cAMP signaling pathway, and transcriptional misregulation in cancer pathway (Fig. 3b).

### Validation by qRT-PCR

**Fig. 4:**
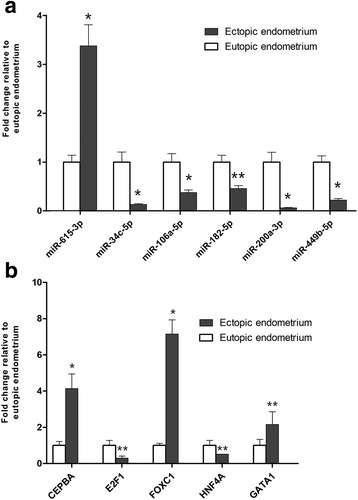
qRT-PCR analysis of the six miRNAs expressions (**a**) and five transcription factors expressions (**b**) in ectopic endometria (*n* = 22) and paired eutopic endometria (*n* = 22). Grey column represents ectopic endometrium; white column represents eutopic endometrium. Significant fold changes in ectopic vs. eutopic endometrium are marked by *, *P* ≤ 0.01; ** *P* ≤ 0.001. The error bar denotes SEM (standard error of the mean)

The qRT-PCR results were consistent with the sequencing results in that all six selected miRNAs (miR-34c-5p, miR-106a-5p, miR-182-5p, miR-200a-3p, miR-449b-5p, and miR-615-3p) and five TFs (CEBPA, FOXC1, E2F1, GATA1, and HNF4A) were differentially expressed with the same trend (up- or down-regulated) of sequencing and reached statistical significance (Fig. 4).

## Discussion

Several microarray studies were conducted to obtain DEMs between EC and EU from women with endometriosis [18, 46, 47], but overlapping data are limited, which suggests that the molecular changes caused by endometriosis might be more complex than we assumed. Additionally, miRNA target prediction methods based on sequence complementarity have been used to annotate the functions of DEMs in these studies, which may contain a high false positive rate and false negative rate [48]. Therefore, the functions of most of the identified DE miRNAs in endometriosis remain unclear due to a lack of definite target genes and confounding downstream validations. Network analysis allows structured grouping of miRNAs, TFs and target genes; thus, network construction is essential for identifying critical molecular changes and understanding their functional roles. To our knowledge, this is the first study to establish concomitant miRNA-TF-gene binary interactions and regulatory networks between the EC and the EU from patients with endometriosis. At first, our study revealed 107 DEMs and 6112 DEGs by sequencing. The large differential expression profiling was refined by PCC to 22 functionally DE miRNAs, 12 TFs and 430 target genes. The network included some well-studied candidate miRNAs and genes (e.g., miR-183-5p, FOXO1, and VEGFA) but also identified many novel objects (e.g., the miR-449 family and miR-106a-363 cluster). Here, we discuss these key regulators in the network, with the hope of providing valuable information for understanding the molecular mechanism of endometriosis.

The highly conserved miR-449 family encoded by the CDC20B gene consists of three members: miR-449a, miR-449b, and miR-449c. Accumulating evidence suggests that the miR-449 family plays an important role in cell proliferation, migration and invasion and might serve as a tumor suppressor in miRNAs [49]. Consistently, miR-449 members were absent or down-regulated in various human malignancies, such as liver cancer [50], breast cancers [51], lung cancer [52], and ovarian clear cell carcinoma [53]. In the current study, we reported the first significant down-regulation of miR-449a, miR-449b-5p, and miR-449c-5p in ectopic endometrial tissue. Considering that endometriosis shares many characteristics with malignant diseases, such as invasion and immigration, it is very likely that the lower expression of miR-449 plays an important role in the pathogenesis of endometriosis. miR-34b and miR-34c are classified into the same cluster and share related functions with miR-449 due to their structural similarities [54, 55]. Much attention has been focused on miR-34 therapeutics for cancer [56, 57]. In addition, miR-34 mimics have been used in several preclinical studies and a phase I clinical trial, and they have shown potential as anticancer therapeutics [58]. Our study identified the down-regulation of miR-34b-3p, which is consistent with the results from Teague et al. [16]. Moreover, we also identified two down-regulated miRNAs, namely, miR-34b-5p and miR-34c-5p, which have been reported by Burney et al. in the eutopic endometrium compared with normal endometrium [17]. Their results suggested that down-regulation of miR-34b and miR-34c was involved in the initiation and maintenance of endometriosis. Given the therapeutic role of miR-34 in cancer and its structural similarity to miR-449 [54], mimics from the miR-449 and miR-34b/c cluster could also be potential miRNA replacement therapies in endometriosis. We therefore suggest that further studies focus on both the miR-34 and miR-449 families by analyzing their function and target genes in endometriosis.

The miR-200 family is another important set of miRNAs that was down-regulated in the present study and has been reported to be reduced in many solid tumors [59]. The miR-200 family contains two groups, miR-200a/b/429 and miR-200c/141, that regulate a cohort of target genes involved in epithelial-mesenchymal transition, metastasis and angiogenesis [60], which are important events in the pathogenesis of endometriosis [61]. Previous studies revealed deregulated miR-200 family members in endometriotic lesions compared with eutopic endometrium [16, 18] and in eutopic endometrium compared with normal endometrium [19]. Circulating miR-200a and miR-141 have also been proposed as candidate biomarkers for endometriosis [62]. The present study is not the first to identify lower levels of miR-141-3p, miR-200a-3p, miR-200b-3p and miR-200c-3p in endometriosis. However, our report confirmed the previous results and identified a series of corresponding genes of these miRNAs, which may be involved in many physical or pathological processes in endometriosis. For example, JAZF1, which is regulated by miR-200b/c, is a proto-oncogene known to be affected by chromosomal translocation in endometrial stromal tumors [63]. In the study by Yotova et al., JAZF1 was hyper-methylated and down-regulated in stromal cells from ovarian ectopic endometria compared with normal endometria [25]. As another example, TP73, which is regulated by miR-200b, is part of the TP53 family and plays a role in the cell cycle, apoptosis and infertility [64], and no previous research has revealed its functions in endometriosis.

miR-363, miR-20b, and miR-106a are transcribed from the miR-106a-363 cluster [65]. All of these miRNAs were down-regulated in ectopic lesions according to our results. Intriguingly, these miRNAs have dual functions either as tumor suppressors or as oncogenic miRNAs. A high level of miR-363-3p suppresses the proliferation of human hepatocellular carcinoma cells [66] and decreases the metastasis of neuroblastoma cells [67], whereas the knock-down of miR-363-3p was reported to suppress carcinogenesis in gastric cancer cells [68]. Li et al. demonstrated that miR-20b acts as an oncogene by contributing to breast tumorigenesis [69], but Hong et al. found that miR-20b was markedly down-regulated and served as a tumor suppressor miRNA in papillary thyroid carcinoma [70]. Li et al. identified increased miR-106a as playing an oncogenic role in pancreatic cancer [71]. In contrast, Zhi et al. reported that miR-106a could inhibit proliferation and promote apoptosis in astrocytoma cells [72]. As no study has previously reported the functions of miR-363-3p, miR-20b and miR-106a in endometriosis, we infer that the decreased expression of these three miRNAs might have tumor suppressive-like functions in the initiation, progression, and metastasis of endometriotic lesions. Additionally, many corresponding target genes, such as ADAMTS family members, which are also known tumor suppressor genes, are down-regulated, consistent with the present study, which confirmed our inference indirectly.

miR-182-5p, which belongs to the miR-183/96/182 cluster, contains the highest node degree in the identified DEMs and shows a 5.3-fold down-regulation in EC versus EU. This finding is consistent with the results obtained from the microarray screening and qRT-PCR validation by Filigheddu et al. [18] Similar to miR-182-5p, miR-183-5p expression in ectopic endometrium was 5.7-fold down-regulated in our study and was confirmed by previously published reports [47, 73]. Additionally, functional analysis indicated that the down-regulation of miR-183 can inhibit apoptosis and enhance the invasive ability of endometrial stromal cells [47]. These findings indicate that aberrant miR-182/183 expression is part of the epigenetic mechanism of pathogenesis and the development of endometriosis.

Based on the integrative regulatory network, a series of TFs also attracted our attention, including FOX family members, GATA family members, and E2F family members, as well as CEBPA, SOX9 and HNF4A. FOX (fork-head box) proteins are a family of transcription factors involved in many pathologic and physiological processes, such as cell growth, proliferation, and longevity [74]. The FOX subfamilies, such as FOXO, FOXC and FOXP, play different roles in different types of diseases [75]. Here, we identified four important FOX subfamily members, FOXO1, FOXC1, FOXL1 and FOXJ1, which cooperatively regulate the integrative networks in endometriosis. FOXO1 serves as an anti-oncogene in various malignancies through diverse mechanisms, such as promoting apoptosis and facilitating DNA repair [76] and was decreased by 3.5-fold in endometriotic tissue in the present study. Previous data showed the same trend with lower FOXO1 levels expressed in endometriosis, which might contribute to an overactive PI3K/AKT pathway, but the underlying mechanisms are not well understood [77]. Additionally, two FBLs, miR-182-5p/FOXO1/NUAK1 and miR-182-5p/FOXO1/SCN9A, were observed and their functions are expected to be explored in the future. In recent years, an elevated expression of FOXC1 has been detected in different tumors, such as esophageal cancer [78], hepatocellular carcinoma [79] and osteosarcoma [80]. The current views on its function and molecular mechanisms reveal that FOXC1 as an oncogene plays multiple roles in cancer progression and metastasis by affecting different targets [78–81]. The present study evaluated FOXC1 expression by high-throughput sequencing and qRT-PCR, and both results indicated that FOXC1 increased approximately sevenfold in EC compared with EU. Furthermore, FOXC1 was identified as a direct target of miR-133b and shares the same targets including EYA4 and LHFP.

GATA1, GATA2 and GATA6 belong to the GATA family, which mainly plays roles in morphogenesis and organogenesis [82]. Our study observed that GATA1 could increase the miR-202-3p expression level and act on 40 targets in the endometriotic lesion. Although the cause of the high expression of GATA1 is not yet clear, Hawkins et al. reported that miR-202-3p was up-regulated in EC compared to normal endometria [19]. Moreover, we suggest that GATA2 and GATA6, as target TFs of the miR-200 family, might be involved in the pathogenesis of endometriosis together with the miR-200 family.

E2F1 is the most important TF in the E2F family, which is known to integrate cell cycle progression with DNA repair, replication, and multiple checkpoints [83]. Interestingly, the function of E2F1 in tumors is a paradox; both positive and negative effects of E2F1 on tumorigenesis have been observed [84]. The present study showed down-regulation of the E2F1 target miR-449 family members, which suggests that their combination possibly initiates endometriosis. E2F2, another important family member of E2F, is also involved in regulating the cell cycle and is associated with cancer growth [85]. A previous study reported that E2F2 can promote the development of liver cancer [86] and can also inhibit the proliferation of prostate cancer cell lines [87]. In our joint analysis, we observed that the down-regulated E2F2 is consistent with a lower expression of miR-196b-5p. Before our research, Abe et al. found that miR-196b-5p was down-regulated in ectopic endometria compared with normal endometria by microarray. Their subsequent functional experiments revealed that miR-196b can inhibit proliferation and induce apoptosis in endometriotic stromal cells [21], which led us to hypothesize that E2F2 might also play important roles in endometriosis by targeting miR-196b.

CEBPA is an important TF controlling hematopoietic differentiation and homeostasis, gene mutation and aberrant expression, which may contribute to hematological system diseases [88]. We observed that highly expressed CEBPA in EC targets 49 corresponding genes and that like FOXO1, was targeted by miR-182-5p. In a recent study, Wang et al. reported that miR-182 can repress CEBPA in hepatocellular carcinoma and is involved in the AKT signaling pathway [89]. This negative correlation between miR-182 and CEBPA is consistent with our result; however, their specific mechanism and involvement in the downstream signaling pathway in endometriosis need to be further confirmed.

Notably, the integrative data revealed that HNF4A targets 12 miRNAs and thus might be a miRNA “hub” in endometriosis. HNF4A is a critical factor in liver function and digestive diseases [90]. The mechanisms of HNF4A and its target miRNAs have not been fully elucidated yet; it is known that HNF4A can directly up-regulate some specific miRNAs to stabilize the hepatocyte phenotype [91]. This unexpected finding requires further validation to identify the underlying mechanism mediated by HNF4A in endometriosis.

GO and KEGG enrichment analysis delineated 42 functional terms and 27 important signaling pathways, most of which are consistent with the current knowledge on endometriosis. For example, the most significant term and pathway are plasma membrane (GO: 0005886) and the PI3K-Akt signaling pathway, which enriched 145 and 17 DEGs, respectively. Ectopic lesions are exposed to a unique peritoneal microenvironment that is characterized by elevated levels of hormones, inflammation, oxidative stress and iron [92]. These abnormal molecules can influence cell survival and regulate cellular functions through binding to cell membrane receptors, and then lead to the subsequent cascading activation of kinase signaling pathways [93]. Therefore, the plasma membrane term and PI3K-Akt signaling pathway are clearly relevant for endometriosis. The PI3K-Akt pathway, which integrates a variety of extracellular signals and regulates various cellular functions, including cell growth, differentiation, transformation and survival, is being investigated as a therapeutic target in other diseases and thus may also represent a target for endometriosis treatment [94, 95]. In addition, our enrichment analysis revealed some novel dysregulated signaling pathways, such as the Rap1 signaling pathway and the retrograde endocannabinoid signaling pathway, and their functions should be further investigated.

Despite our novel results, the limitations of this study should also be mentioned. First, the number of sequenced samples was relatively inadequate, and only non-hormonal treated patients with moderate to severe stage disease within the secretory phase were enrolled. Endometriosis is a complex disease that is influenced by multiple factors, such as the menstrual cycle, CA125 levels and various subtypes. These differences might impact the expression profile. Although normalized samples are helpful to determine the key pathogenic molecules, our study was most likely underpowered in discovering DEMs and DEGs with subtle expression differences due to the limited sample size. Second, the present results are preliminary and descriptive. Integrative analysis of paired miRNA and mRNA profiling data cannot entirely exclude false positive results. In addition, only some of the identified miRNAs and TFs were validated by qRT-PCR. Therefore, other experiments, such as cross-linking immunoprecipitation or functional experimental validation, are needed to holistically validate the interactions identified by our method. Furthermore, the positive findings that were not included in the network might contain valuable information and are worthy of further discussion. Third, various computational methods have been devised to measure the miRNA-mRNA regulatory relationships in recent years, such as correlation analysis, regression models, Bayesian network learning and causal inference techniques [96]. However, which one is the best method remains an open question in practical use. Here, we used the typical Pearson correlation coefficient to analyze the relationship between DEMs and DEGs, which might lead to some differences from other methods and an inevitable loss of information.

## Conclusion

In summary, we provide the first characterization of miRNA-TF-gene co-expression network in paired miRNA and mRNA expression profiling of endometriosis. By establishing the regulatory network in miRNA and mRNA expression profiling, our present study confirms and significantly extends the results of prior studies, thereby defining some crucial miRNAs, TFs and genes involved in the pathogenesis of endometriosis. The identified interactions could also have implications toward targeted therapeutic strategies for endometriosis. Further in-depth functional studies are encouraged to confirm our results.

## Additional files

Additional file 1: Clinical characteristics of 30 enrolled patients with ovarian endometriosis. (DOCX 19 kb)

Additional file 2: Supplementary methods introduction for small RNA-seq and mRNA seq. (DOCX 21 kb)

Additional file 3: Primers had been used in qRT-PCR (DOCX 14 kb)

Additional file 4: Summary of the Small RNA sequencing data after filtering and mapping (DOCX 15 kb)

Additional file 5: List of 107 differentially expressed miRNAs in ectopic endometria compared with eutopic endometria (DOCX 17 kb)

Additional file 6: Summary of the mRNA sequencing data after filtering and mapping (DOCX 16 kb)

Additional file 7: Top 25 up-regulated and down-regulated mRNAs in ectopic endometria compared with paired eutopic endometria in ovarian endometriosis. (DOCX 15 kb)

## Abbreviations

BP: Biological process
CC: GO cellular component
DEG: Differentially expressed gene
DEM: Differentially expressed miRNA
EC: Ectopic endometrium
EU: Eutopic endometrium
FBL: Feed-back loop
FC: Fold change
FDR: False discovery rate
FFL: Feed-forward loop
GO: Gene Ontology
KEGG: Kyoto Encyclopedia of Genes and Genomes
MF: Molecular function
miRNA: microRNA
mRNA: messenger RNA
PCC: Pearson correlation coefficient
qRT-PCR: Quantitative reverse transcription-polymerase chain reaction
r-AFS: Revised American fertility society
RPKM: Reads per kilobase of one gene per million reads
RPM: Reads per million total reads
small RNA-seq: Small RNA sequencing
TF: Transcription factors
