# Supplementary material for: Integration analysis of microRNA and mRNA paired expression profiling identifies deregulated microRNA-transcription factor-gene regulatory networks in ovarian endometriosis
Source: Reprod Biol Endocrinol. 2018 Jan 22;16:4. doi: 10.1186/s12958-017-0319-5 (PMC5776778; doi:10.1186/s12958-017-0319-5)
Supplement: Supplementary file 1 — Clinical characteristics of 30 enrolled patients with ovarian endometriosis. (DOCX 19 kb) [file 12958_2017_319_MOESM1_ESM.docx]

**Additional file 1:** Clinical characteristics of 30 enrolled patients with ovarian endometriosis.

| **Patient ID** | **Group** | **Age** | **BMI** | **History of gestation** | **r-AFS stage** | **Other diagnosis** | **CA125 (u/ml)** |
| --- | --- | --- | --- | --- | --- | --- | --- |
| EC/EU1 | Sequencing set | 23 | 22 | G0P0 | III | None | 25.6 |
| EC/EU2 | Validation set | 38 | 21 | G2P1 | IV | DIE | 50.7 |
| EC/EU3 | Validation set | 32 | 18 | G3P1 | III | Myoma | 24.3 |
| EC/EU4 | Validation set | 37 | 20 | G1P1 | IV | None | 86.6 |
| EC/EU5 | Sequencing set | 31 | 21 | G2P1 | IV | None | 50.7 |
| EC/EU6 | Sequencing set | 25 | 18 | G0P0 | III | None | 86.9 |
| EC/EU7 | Sequencing set | 26 | 20 | G0P0 | IV | None | 28.04 |
| EC/EU8 | Validation set | 30 | 19 | G0P0 | III | None | 43.8 |
| EC/EU9 | Validation set | 38 | 23 | G2P1 | IV | PE | 140.6 |
| EC/EU10 | Validation set | 27 | 22 | G2P0 | III | None | 73.57 |
| EC/EU11 | Sequencing set | 25 | 21 | G1P0 | III | None | 23.2 |
| EC/EU12 | Validation set | 50 | 21 | G5P3 | IV | None | 165.4 |
| EC/EU13 | Validation set | 31 | 16 | G2P1 | IV | None | 61.7 |
| EC/EU14 | Validation set | 35 | 24 | G3P2 | III | Hydrosalpinx | N/A |
| EC/EU15 | Validation set | 40 | 18 | G2P1 | IV | None | 44.8 |
| EC/EU16 | Validation set | 29 | 21 | G0P0 | III | None | 21.4 |
| EC/EU17 | Validation set | 33 | 18 | G4P1 | III | Myoma | 40.2 |
| EC/EU18 | Sequencing set | 43 | 17 | G1P1 | III | None | 47.3 |
| EC/EU19 | Validation set | 31 | 21 | G0P0 | IV | None | 52.9 |
| EC/EU20 | Validation set | 38 | 19 | G3P2 | III | None | 46.2 |
| EC/EU21 | Sequencing set | 25 | 17 | G0P0 | IV | PE | 28.0 |
| EC/EU22 | Validation set | 28 | 22 | G0P0 | IV | None | 30.5 |
| EC/EU23 | Validation set | 45 | 21 | G1P1 | III | None | 181.9 |
| EC/EU24 | Validation set | 33 | 24 | G1P1 | III | None | N/A |
| EC/EU25 | Sequencing set | 37 | 19 | G0P0 | IV | None | 116.6 |
| EC/EU26 | Validation set | 26 | 20 | G4P0 | III | None | 39.5 |
| EC/EU27 | Validation set | 26 | 19 | G1P0 | III | Mesosalpinx cyst | 40.5 |
| EC/EU28 | Validation set | 31 | 21 | G3P1 | IV | DIE | N/A |
| EC/EU29 | Validation set | 33 | 21 | G0P0 | III | Myoma | 56.1 |
| EC/EU30 | Validation set | 32 | 23 | G2P0 | III | Ovarian simple cyst | 68.5 |

Note: Samples from patients 1, 5, 6, 7, 11, 18, 21, 25 were used in the high-throughput sequencing, and the rest of patients were used in the qRT-PCR validation. Other diagnosis: diagnosis other than ovarian endometriosis. BMI= body mass index; r-AFS=revised American Fertility Society; N/A=not available; DIE=deep infiltrating endometriosis; PE=peritoneal endometriosis.
