# Supplementary material for: Integration analysis of microRNA and mRNA paired expression profiling identifies deregulated microRNA-transcription factor-gene regulatory networks in ovarian endometriosis
Source: Reprod Biol Endocrinol. 2018 Jan 22;16:4. doi: 10.1186/s12958-017-0319-5 (PMC5776778; doi:10.1186/s12958-017-0319-5)
Supplement: Supplementary file 2 — Supplementary methods introduction for small RNA-seq and mRNA seq. (DOCX 21 kb) [file 12958_2017_319_MOESM2_ESM.docx]

**Additional file 2:** Supplementary methods introduction for small RNA-seq and mRNA seq.

Part 1. Data analysis for small RNA sequencing

1. Quality control

Raw data were processed with Python scripts to ensure the quality of the data used in further analysis. The adopted filtering criteria were as follows:

(1) Remove reads without 3’ adapters. The contaminated reads for adapters were defined such that the read bases contained more than 5 bp of adapter sequences.

(2) Remove reads without insert fragments. After removing the 5’ and 3’ adapter of reads, no bases existed.

(3) Remove reads with too much poly A/T (The total content of A/T was more than 80% or continuous A/T exceed than 20%).

(4) Remove reads with lengths outside a certain range. miRNA length distribution line should have a length of 22 bases.

(5) Remove low quality reads. Low quality reads were defined such that the number of read bases whose phred quality value was less than or equal to 19 accounted for more than 15%.

(6) Remove the reads that contained N bases more than 5% of the total. The clean data were filtered, which were statistically significant for the quality and data quantity, including Q30 statistics and data content statistics.

2. Alignment

Reference gene and genome annotation files were downloaded from Ensemble (GRCh37/Hg19) (http: //www.ensembl.org/index.) using Bowtie (1.0.1) to build the reference genome library, and then the clean data were mapped to the reference genome. The software Bowtie 1 was used to accurately and rapidly align short reads (mostly shorter than 50 bp) from a large genome.

3. miRNA Identification

The basic idea of known miRNA identification is to get an overlap (100%) between the genome location of mapping reads and the genome location of reference miRNA. If genome annotation data can be found in miRBase (Release 21), we need to get the overlap by Bedtools (<http://bedtools.readthedocs.io/en/latest/>) software. Otherwise, we first map the reference miRNA to the reference genome to obtain location information and then get the overlap.

4. Quantitation of miRNA expression levels
For every sample, the count and RPM of miRNAs were collected. RPM (reads per million total reads) values can be considered a normalized count of samples and can be directly used in inter-library comparison.

5. Differential miRNA expression analysis
DEGseq (v1.18.0) was used for differential gene expression analysis. Under the assumption that the number of reads derived from a miRNA follows a binomial distribution, DEGseq is proposed based on MA-plot and is widely used for differential gene expression analysis. A P value could be assigned to each gene and adjusted by Benjamini and Hochberg’s approach to control the false discovery rate. miRNAs with q<0.05 and |log2_ratio|≥1 are identified as differentially expressed miRNAs (DEMs).

Part 2. Introduction of methods of mRNA analysis

1. Quality Control

Raw data are processed with Perl scripts to ensure the quality of data used in further analysis. The adopted filter criteria are:

(1) Filter out adaptor-polluted reads. Reads containing more than 5 adapter-polluted bases are regarded as adaptor-polluted reads and are filtered out.

(2) Filter out low-quality reads. Reads with a number of bases whose phred quality value was no more than 19, accounting for more than 15%, are considered low-quality reads and are filtered out.

(3) Filter out reads with the number of N bases accounting for more than 5%.

Clean data are obtained after filtering, and statistical analyses are performed on their quantity and quality, including Q30 statistics, data quantity statistics and base content statistics.

2. Mapping sequencing reads to reference genomes

The reference genomes and annotation file were downloaded from the ENSEMBL database (GRCh37) (http://grch37.ensembl.org/index.html). Bowtie 2 (v2.2.3) was used to build the genome index, and clean data were mapped to the reference genome using TopHat (v2.0.12).

3. Quantitation of gene expression

Read counts for each gene in each sample were counted by HTSeq (v0.6.0), and RPKM (Reads Per Kilobase Million Mapped Reads) were then calculated to represent the expression level of genes in each sample.

4. Differential Expression Analysis

DEGseq (v1.18.0) was used for the differential expression analysis of two samples with replicates. A P value could be assigned to each gene and adjusted by Benjamini and Hochberg’s approach. Genes with q≤0.05 and |log2_ratio|≥1 are identified as differentially expressed genes (DEGs).

More detailed information is available on request.
