# Supplementary material for: Integration analysis of microRNA and mRNA paired expression profiling identifies deregulated microRNA-transcription factor-gene regulatory networks in ovarian endometriosis
Source: Reprod Biol Endocrinol. 2018 Jan 22;16:4. doi: 10.1186/s12958-017-0319-5 (PMC5776778; doi:10.1186/s12958-017-0319-5)
Supplement: Supplementary file 3 — Primers had been used in qRT-PCR (DOCX 14 kb) [file 12958_2017_319_MOESM3_ESM.docx]

**Additional file 3:** Primers had been used in qRT-PCR

| Primers |  | Sequences (5’ to 3’) |
| --- | --- | --- |
| miR-34c-5p | Forward | AGGCAGTGTAGTTAGCTGATTGC |
| miR-106a-5p | Forward | AAAAGTGCTTACAGTGCGGGTA |
| miR-182-5p | Forward | TGGCAATGGTAGAACTCACACTC |
| miR-200a-3p | Forward | TGGCACTGTCTGGTAACGATG |
| miR-449b-5p | Forward | ACACTCCAGCTGGGAGGCAGTGCAG |
| miR-615-3p | Forward | CCGAGCCTGGGTCTCCCT |
| U6 | Forward | CTCGCTTCGGCAGCACA |
|  | Reverse | AACGCTTCACGAATTTGCGT |
| CEBPA | Forward | AACACGAAGCACGATCAGTCC |
|  | Reverse | CTCATTTTGGCAAGTATCCGA |
| FOXC1 | Forward | GCCAGCAGCAGAACTTCCA |
|  | Reverse | GAGAGTTGTTCAAGCCGATCCT |
| E2F1 | Forward | GCAGAGCAGATGGTTATGGTGAT |
|  | Reverse | GGAGATGATGGTGGTGGTGACA |
| GATA1 | Forward | TGGAGACTTTGAAGACAGAGCGGCTGAG |
|  | Reverse | GAAGCTTGGGAGAGGAATAGGCTGCTGA |
| HNF4A | Forward | ACCTGCTGCCATCCAACCA |
|  | Reverse | CCTCCTACATCTGCCATGAACA |
| GAPDH | Forward | CTGGGCTACACTGAGCACC |
|  | Reverse | AAGTGGTCGTTGAGGGCAATG |
