# Supplementary material for: Integration analysis of microRNA and mRNA paired expression profiling identifies deregulated microRNA-transcription factor-gene regulatory networks in ovarian endometriosis
Source: Reprod Biol Endocrinol. 2018 Jan 22;16:4. doi: 10.1186/s12958-017-0319-5 (PMC5776778; doi:10.1186/s12958-017-0319-5)
Supplement: Supplementary file 4 — Summary of the Small RNA sequencing data after filtering and mapping (DOCX 15 kb) [file 12958_2017_319_MOESM4_ESM.docx]

**Additional file 4**: Summary of the Small RNA sequencing data after filtering and mapping

| Sample | Total Raw Reads | Clean Reads Q30 (%) | Total Clean Reads | Perfect Match Reads | Perfect Match Rate (%) |
| --- | --- | --- | --- | --- | --- |
| EC1 | 13091130 | 97.75 | 11113652 | 9060085 | 82.14 |
| EC5 | 13877400 | 97.74 | 11669347 | 9195679 | 80.36 |
| EC6 | 13100843 | 97.7 | 11384226 | 8952552 | 78.93 |
| EC7 | 13338186 | 97.83 | 11547722 | 9472031 | 82.53 |
| EC11 | 13216079 | 97.76 | 11083699 | 9077425 | 83.07 |
| EC18 | 14893608 | 97.85 | 11049638 | 8868453 | 81.96 |
| EC21 | 12739484 | 98.68 | 11437953 | 8892180 | 77.95 |
| EC25 | 14603314 | 97.56 | 11956147 | 9620920 | 82.46 |
| EU1 | 13601954 | 97.38 | 11200787 | 8860946 | 79.68 |
| EU5 | 13465861 | 97.7 | 11204683 | 9075091 | 82.49 |
| EU6 | 13348597 | 97.74 | 11542385 | 9479403 | 82.58 |
| EU7 | 12878315 | 97.7 | 11004948 | 9025354 | 82.67 |
| EU11 | 13484505 | 97.75 | 11533450 | 9314241 | 81.25 |
| EU18 | 13235598 | 98.01 | 11328213 | 9285155 | 82.84 |
| EU21 | 13792820 | 97.68 | 11862717 | 9757106 | 83.03 |
| EU25 | 13665889 | 97.7 | 11500813 | 9448193 | 83.42 |
