# Supplementary material for: Integration analysis of microRNA and mRNA paired expression profiling identifies deregulated microRNA-transcription factor-gene regulatory networks in ovarian endometriosis
Source: Reprod Biol Endocrinol. 2018 Jan 22;16:4. doi: 10.1186/s12958-017-0319-5 (PMC5776778; doi:10.1186/s12958-017-0319-5)
Supplement: Supplementary file 5 — List of 107 differentially expressed miRNAs in ectopic endometria compared with eutopic endometria (DOCX 17 kb) [file 12958_2017_319_MOESM5_ESM.docx]

**Additional file 5:** List of 107 differentially expressed miRNAs in ectopic endometria compared with eutopic endometria

| miRNA | Fold Change | miRNA | Fold Change |
| --- | --- | --- | --- |
| miR-514b-3p | 330.31 | miR-34c-3p | -25.24 |
| miR-510-5p | 123.89 | miR-3616-3p | -25.03 |
| miR-509-3-5p | 58.5 | miR-200a-5p | -24.87 |
| miR-514b-5p | 49.43 | miR-200b-5p | -24.5 |
| miR-508-5p | 42.46 | miR-885-3p | -24.27 |
| miR-202-5p | 15.21 | miR-187-3p | -23.11 |
| miR-6502-5p | 10.04 | miR-203b-3p | -22.8 |
| miR-133b | 9.16 | miR-34b-5p | -22.17 |
| miR-216a-5p | 7.37 | miR-573 | -19.48 |
| miR-217 | 6.83 | miR-934 | -17.08 |
| miR-4649-3p | 6.64 | miR-200b-3p | -16.72 |
| miR-133a-3p | 6.56 | miR-6718-5p | -16.61 |
| miR-216a-3p | 5.6 | miR-34c-5p | -15.91 |
| miR-708-5p | 5.29 | miR-196b-3p | -14.15 |
| miR-1243 | 5.05 | miR-196b-5p | -13.72 |
| miR-653-3p | 4.86 | miR-3664-3p | -13.9 |
| miR-615-3p | 4.36 | miR-876-3p | -12.96 |
| miR-202-3p | 4.2 | miR-34b-3p | -12.5 |
| miR-223-3p | 3.71 | miR-876-5p | -12.38 |
| miR-223-5p | 3.68 | miR-183-3p | -11.95 |
| miR-134-3p | 3.54 | miR-10a-3p | -11.67 |
| hsa-let-7a-2-3p | 3.5 | miR-182-3p | -11.14 |
| miR-874-5p | 3.43 | miR-4636 | -10.92 |
| miR-708-3p | 3.42 | miR-4724-5p | -10.91 |
| miR-592 | 3.23 | miR-873-5p | -9.2 |
| miR-539-5p | 3.12 | miR-10a-5p | -9.2 |
| miR-145-3p | 3.05 | miR-203a-3p | -8.54 |
| miR-127-3p | 3.02 | miR-675-5p | -7.78 |
| miR-656-3p | 2.87 | miR-675-3p | -6.71 |
| miR-143-5p | 2.84 | miR-33b-3p | -6.16 |
| miR-382-5p | 2.75 | miR-3116 | -5.78 |
| miR-493-5p | 2.66 | miR-183-5p | -5.72 |
| miR-154-5p | 2.66 | miR-190b | -5.5 |
| miR-100-5p | 2.61 | miR-182-5p | -5.34 |
| miR-539-3p | 2.45 | miR-203a-5p | -5.23 |
| miR-381-3p | 2.43 | miR-1266-5p | -5.17 |
| miR-134-5p | 2.39 | miR-30d-3p | -4.8 |
| miR-127-5p | 2.38 | miR-556-5p | -4.64 |
| miR-136-3p | 2.26 | miR-345-5p | -4.35 |
| miR-214-3p | 2.16 | miR-20b-5p | -4.16 |
| miR-22-3p | 2.06 | miR-30d-5p | -3.93 |
| miR-375 | -154.52 | miR-224-5p | -3.82 |
| miR-449c-5p | -115.64 | miR-106a-5p | -3.72 |
| miR-449b-5p | -114.14 | miR-3614-5p | -3.48 |
| miR-767-5p | -93.7 | miR-30b-3p | -3.36 |
| miR-449a | -70.83 | miR-561-5p | -3.02 |
| miR-200c-5p | -67.42 | miR-33b-5p | -3.02 |
| miR-449b-3p | -56.95 | miR-363-3p | -2.92 |
| miR-141-3p | -56.69 | miR-30b-5p | -2.67 |
| miR-141-5p | -36.13 | miR-455-3p | -2.53 |
| miR-200a-3p | -35.83 | miR-425-5p | -2.42 |
| miR-3616-5p | -32.37 | miR-30a-5p | -2.4 |
| miR-873-3p | -29.52 | miR-17-5p | -2.38 |
| miR-200c-3p | -27.15 |  |  |
