# Supplementary material for: Integration analysis of microRNA and mRNA paired expression profiling identifies deregulated microRNA-transcription factor-gene regulatory networks in ovarian endometriosis
Source: Reprod Biol Endocrinol. 2018 Jan 22;16:4. doi: 10.1186/s12958-017-0319-5 (PMC5776778; doi:10.1186/s12958-017-0319-5)
Supplement: Supplementary file 6 — Summary of the mRNA sequencing data after filtering and mapping (DOCX 16 kb) [file 12958_2017_319_MOESM6_ESM.docx]

**Additional file 6:** Summary of the mRNA sequencing data after filtering and mapping

| Sample | Total Raw Reads Number | Total Q30(%) | Total Clean Reads Number | Mapped Reads | UniqueMap Reads | MultiMap Reads |
| --- | --- | --- | --- | --- | --- | --- |
| EC1 | 119,966,674 | 94.81 | 118,535,568 | 109,149,775 | 107,379,412 | 1,770,363 |
| EC5 | 98,991,040 | 94.51 | 97,468,196 | 90,454,519 | 89,284,173 | 1,170,346 |
| EC6 | 108,126,726 | 94.74 | 106,280,562 | 99,658,282 | 98,058,541 | 1,599,741 |
| EC7 | 124,675,828 | 94.75 | 123,109,860 | 115,546,897 | 113,967,901 | 1,578,996 |
| EC11 | 112,543,742 | 94.9 | 110,712,030 | 100,840,452 | 99,338,951 | 1,501,501 |
| EC18 | 115,496,112 | 94.67 | 113,551,876 | 104,913,818 | 103,430,961 | 1,482,857 |
| EC21 | 115,216,278 | 93.92 | 112,332,164 | 103,358,698 | 101,757,333 | 1,601,365 |
| EC25 | 95,882,152 | 94.82 | 94,596,564 | 86,811,645 | 85,394,613 | 1,417,032 |
| EU1 | 108,063,442 | 94.38 | 106,300,690 | 99,490,109 | 98,103,400 | 1,386,709 |
| EU5 | 115,796,574 | 94.93 | 114,245,924 | 108,073,952 | 106,633,173 | 1,440,779 |
| EU6 | 88,538,384 | 94.76 | 87,285,608 | 81,742,080 | 80,563,047 | 1,179,033 |
| EU7 | 125,048,606 | 94.97 | 123,155,952 | 116,419,348 | 114,821,450 | 1,597,898 |
| EU11 | 112,650,384 | 94.35 | 110,637,684 | 102,884,399 | 101,501,805 | 1,382,594 |
| EU18 | 89,731,074 | 94.42 | 88,482,524 | 83,389,862 | 82,191,074 | 1,198,788 |
| EU21 | 105,616,842 | 94.57 | 104,296,544 | 97,924,454 | 96,502,461 | 1,421,993 |
| EU25 | 112,190,626 | 94.68 | 110,717,296 | 103,453,282 | 102,134,657 | 1,318,625 |
