# Supplementary material for: Integration analysis of microRNA and mRNA paired expression profiling identifies deregulated microRNA-transcription factor-gene regulatory networks in ovarian endometriosis
Source: Reprod Biol Endocrinol. 2018 Jan 22;16:4. doi: 10.1186/s12958-017-0319-5 (PMC5776778; doi:10.1186/s12958-017-0319-5)
Supplement: Supplementary file 7 — Top 25 up-regulated and down-regulated mRNAs in ectopic endometria compared with paired eutopic endometria in ovarian endometriosis. (DOCX 15 kb) [file 12958_2017_319_MOESM7_ESM.docx]

**Additional file 7:** Top 25 up-regulated and down-regulated mRNAs in ectopic endometria compared with paired eutopic endometria in ovarian endometriosis.

| Official symbol | Fold change | Official symbol | Fold change |
| --- | --- | --- | --- |
| IGHV3-49 | 3253.41 | FXYD4 | -1024.16 |
| ARX | 1654.49 | SCGB1D4 | -874.29 |
| MIR202HG | 1254.70 | CWH43 | -745.57 |
| NR5A1 | 1215.97 | RP11-711K1.7 | -657.88 |
| COL10A1 | 986.52 | FUT9 | -647.91 |
| IGLV3-21 | 983.25 | MMP26 | -645.85 |
| PNOC | 972.74 | BARX2 | -641.16 |
| CCL11 | 849.69 | TM4SF4 | -562.01 |
| DLK1 | 847.08 | SCGB2A2 | -507.56 |
| ITLN1 | 765.66 | DLGAP1-AS3 | -496.94 |
| IGKV1-27 | 760.68 | PLA2G4F | -442.05 |
| IGKV1D-16 | 748.64 | B4GALNT2 | -410.99 |
| IGFL2 | 740.21 | SLC30A2 | -364.48 |
| IGHV1-69-2 | 714.47 | GAST | -357.5 |
| IGKV1-16 | 706.08 | RP11-794P6.6 | -337.08 |
| POU6F2 | 698.89 | MUC15 | -331.62 |
| SYT4 | 681.61 | SLC5A1 | -325.59 |
| HS3ST2 | 672.15 | TMEM196 | -321.76 |
| IGKV2D-29 | 661.70 | SLC25A48 | -319.94 |
| IGLV1-47 | 649.39 | LDLRAD1 | -311.36 |
| IGLV7-46 | 631.45 | SCGB1D2 | -295.77 |
| IGHV4-31 | 593.59 | CRISP3 | -284.4 |
| IGLC3 | 591.59 | SYT13 | -280.42 |
| IGLV10-54 | 578.05 | PLA2G4E | -270.75 |
| CYP19A1 | 532.78 | PSG9 | -261.7 |
